# Slowly absorbable suture for fascial defect closure in open incisional hernia mesh-repair is associated with decreased long-term recurrence: a nationwide cohort study

**DOI:** 10.1007/s10029-026-03585-y

**Published:** 2026-02-26

**Authors:** Mads Marckmann, Nadia A. Henriksen, Mette W. Christoffersen, Kristian S. Kiim

**Affiliations:** 1 Digestive Disease Center, Bispebjerg Hospital, University of Copenhagen, Bispebjerg Bakke 26, Copenhagen, 2400 Denmark; 2 Department of Surgery, Zealand University Hospital Koege, Køge, Denmark; 3 Department of Surgery and Transplantation, Rigshospitalet, Copenhagen University Hospital, Copenhagen, Denmark

**Keywords:** Hernia repair, Hernia recurrence, Reoperation, Incisional hernia, Mesh repair, Suture type

## Abstract

**Purpose:**

Recurrence after incisional hernia repair is an important outcome measure with rates still ranging high. For open incisional hernia repair a mesh-based technique with fascial defect closure is recommended, but there is no evidence supporting the choice of suture used for the defect closure. Slowly absorbable suture has been advised as reducing the risk of primary incisional hernia formation after abdominal surgery, but whether this applies as best choice compared to non-absorbable suture in open incisional hernia repair with mesh is undetermined.

**Method:**

This was a nationwide registry study with a 100% follow-up from 2007 to 2022. Eligibility criteria were elective open incisional hernia surgery, mesh-based technique, and fascial defect closure with slowly absorbable or non-absorbable suture. The 5-year cumulative incidence of reoperation for hernia recurrence was determined. Confounders were included in multivariate regression analyses.

**Results:**

A total of 3393 patients were included. Mean (sd) age was 60.6 (13) years and 50% were females. Mean horizontal defect size was 6.4 (4.7) cm and 1900 (56%) patients had vertical incisions. Incidence of 90-day surgical reintervention was 143 (4.2%). Median (IQR) follow-up was 3.5 (1.6–3.8) years, and 249 (7.3%) patients underwent operation for recurrence. Cox regression analysis showed that non-absorbable suture was associated with a significantly increased risk of operation for recurrence compared to slowly absorbable (HR 1.33, CI 1.01–1.76, *P* = 0.043). Type of suture was not associated with increased risk of 90-day reoperation.

**Conclusion:**

Using a slowly absorbable suture for fascial closure is associated with a decreased risk of long-term hernia recurrence compared to non-absorbable suture after open incisional hernia mesh-repair.

## Introduction

The development of incisional hernias is multifactorial, involving both intrinsic and extrinsic factors. These range from endogenous collagen composition and genetic connective tissue disorders to lifestyle-related conditions that impair wound healing [1]. Surgical technique is also critical. Israelsson’s 4:1 suture-to-wound length ratio and low-tension closure are widely accepted principles associated with improved perfusion and reduced risk of incisional hernia after laparotomy [2–4].

Guidelines for primary and incisional hernia repair recommend fascial closure with slowly absorbable sutures. However, most supporting evidence is derived from heterogeneous data on laparotomy closures, and the impact of suture type on recurrence after open mesh repair of incisional hernias remains unclear [5, 6]. Current international recommendations advocate for continuous small-bite suturing with slowly absorbable material, as this reduces the risk of incisional hernia compared to rapidly absorbable sutures [6]. The rationale is biomechanical: rapidly absorbable sutures retain only ~ 25% of their tensile strength at four weeks and are fully resorbed within 8–10 weeks, while fascial healing requires much longer. In contrast, slowly absorbable sutures maintain > 50% of their strength at six weeks and are resorbed after 6–8 months, better matching the prolonged timeline of fascial recovery, which can take up to one year [7–9].

When compared with non-absorbable sutures, slowly absorbable sutures offer advantages not necessarily in recurrence reduction, but in fewer complications such as pain, fistula tracts, and suture sinuses [10–13]. Historically, before the widespread adoption of mesh-based repair, non-absorbable sutures were the standard choice, which can explain the continued use in some mesh-based techniques today [14].

Despite these preventive strategies, the incidence of incisional hernia remains high ~ 13% two years after surgery [15]. While retromuscular mesh-based repair with fascial closure is the recommended treatment for incisional hernias, it remains uncertain whether the choice of suture material for fascial closure influences long-term recurrence rates—a key outcome after incisional hernia repair [6].

This study aimed to compare slowly absorbable and non-absorbable sutures in open incisional hernia mesh repair including two hypotheses:

1. Suture type is of minor importance as the repair mechanism and endurance also rely on the mesh implantation.
2. Slowly absorbable sutures reduce long-term risk of compared to non-absorbable sutures, which are associated with higher risk of pain, suture sinus, and fistula formation.

## Methods

This was a nationwide database study with nearly 100% follow-up accounting for theoretical loss from errors or changes in patient identifiers. The Danish Ventral Hernia Database is a registry with compulsory data registration and is linked to the Danish National Patients Registry, which holds comprehensive information on health care admissions, comorbidities, and medical treatments including operation coding [16, 17].

### Inclusion criteria

This study included patients who underwent open incisional hernia repair with mesh and fascial closure using either slowly absorbable or non-absorbable sutures over a 15-year-period (2007–2022).

### Variables

The primary outcome was the 5-year cumulative incidence of hernia recurrence, defined as operation for recurrence. The secondary outcome was reoperation for complication within 90 days after the index hernia repair. Other variables included for analysis were (1) demographics: age (years), sex assigned at birth (female/male), Charlson Comorbidity Index (CCI, categorized 0;1;2;>2), (2) intraoperative factors: hernia defect size (cm, vertical/horizontal), orientation of incision (vertical, horizontal, other), mesh width (cm), mesh localization (onlay, retromuscular, intraperitoneal, other); and (3) postoperative factors: readmission due to any cause within 90 days after surgery (yes/no) and reoperation within 90 days after surgery (yes/no) [18].

### Statistics

Categorial data were presented as numbers (percentages), and continuous data were shown as mean (standard deviation, SD) or median (interquartile range, IQR). Chi-square tests or the Mann-Whitney tests were used for data comparison across the groups. P-values < 0.05 were deemed statistically significant. Absolute risks (equal to cumulative incidences) of operation for hernia recurrence were estimated using the Kaplan-Meier method. Multivariate analysis was performed using a cox proportional hazards regression model and multiple logistic regression for the outcomes of recurrence and 90-day reoperation, respectively. Selection of the variables included in each analysis was based on the literature. Long-term risk of recurrence and 90-day risk of reoperation were adjusted for sex, age, CCI, type of index surgery (primary incisional or recurrent incisional), orientation of incision, horizontal defect size, mesh position, and type of suture. The statistics were performed using R software version 4.0.2 (R Foundation for Statistical Computing, Vienna, Austria).

### Ethics

The study was approved by the Danish Data Protection Agency (REG-138–2018) and the Danish Hernia Database. The study was reported according to the STROBE statement for cohort studies [19].

## Results

**Fig. 1:**
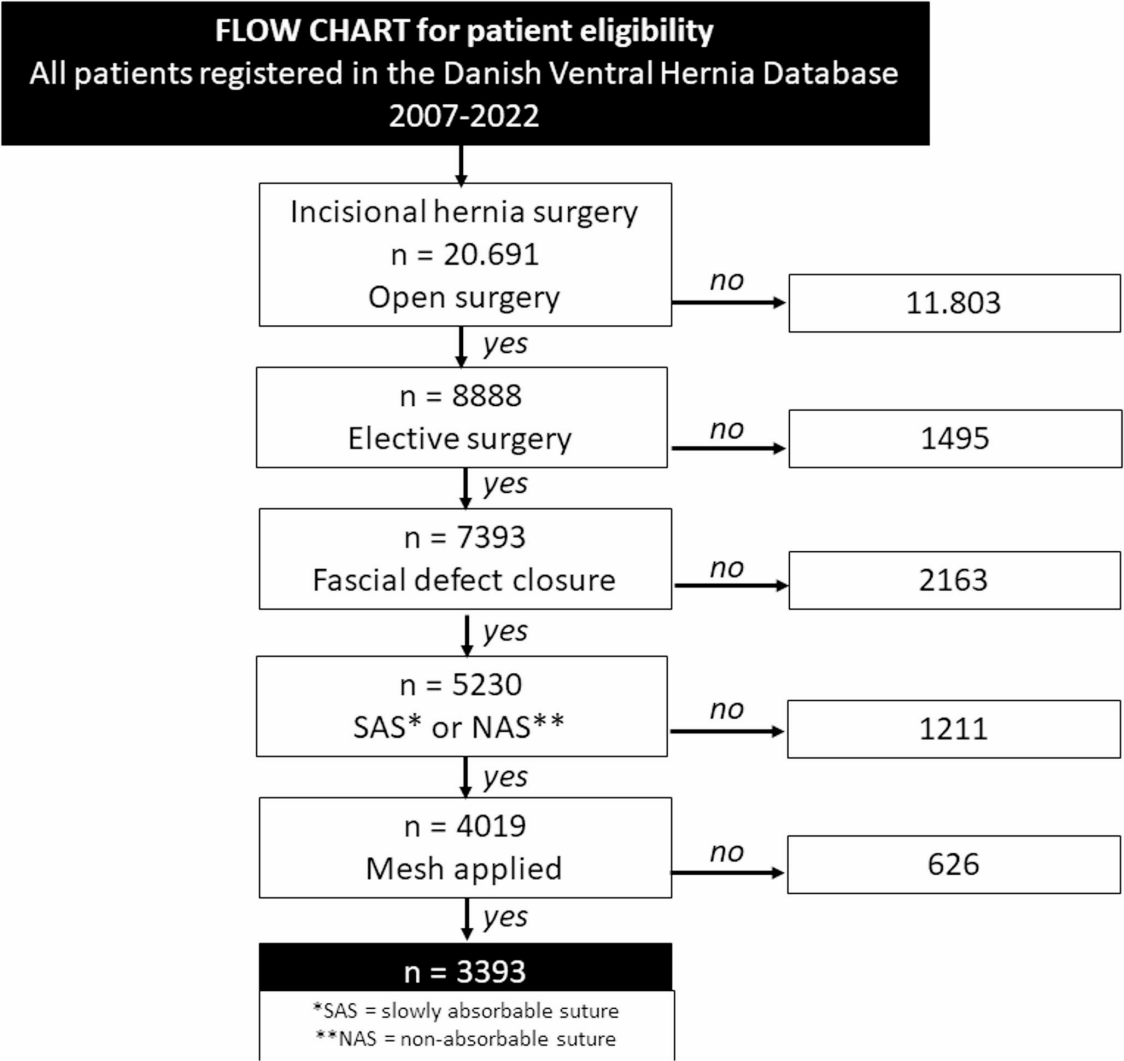
Flowchart

During the study period 20,691 patients underwent incisional hernia repair. Of these 7,393 were open and elective, and after screening for remaining criteria a final census of 3,393 was achieved (see flowchart, Fig. 1). Median (IQR) number of days to follow-up was 1270 (590–1386). A total of 249 (7.3%) patients underwent operation for recurrence. Mean(sd) age of the included patients was 60.6 (13) years and sex distribution (assigned at birth) were balanced (F: 49.7%). Mean(sd) horizontal hernia defect size was 6.4 (4.7) cm and most incisions were vertical (n 1900 = 56%). Slowly absorbable suture was used in 51.2% of the cases. Predominantly, meshes were placed in the retromuscular position (61.4%), other positions were also used, e.g. onlay (30%). Within 90 days after surgery, 627 (18.5%) were readmitted and of the entire cohort 143 (4.2%) underwent reoperation. See Table 1 for characteristics and outcomes according to type of suture.

**Table 1:** Baseline characteristics and outcomes according to type of suture (*CCI = Charlson comorbidity Index*)

Variable		Slowly absorbable (*n* = 1,736)	Non-absorbable (*n* = 1,657)	*P*
Age	mean (sd)	61.5 (12.5)	59.6 (13.3)	< 0.001
Sex	F	829 (47.8)	858 (51.8)	0.021
	M	907 (52.2)	799 (48.2)	
CCI	0	570 (32.8)	668 (40.3)	< 0.001
	1	273 (15.8)	271 (16.4)	
	2	391 (22.5)	368 (22.2)	
	> 2	502 (28.9)	350 (21.1)	
Horizontal defect size (cm)	mean (sd)	8 (4.6)	4.8 (4.2)	< 0.001
Vertical defect size (cm)	mean (sd)	10.7 (6.6)	5.9 (6.5)	< 0.001
Mesh width (cm)	0–9.99.99	199 (11.5)	725 (43.7)	< 0.001
	10- <15	384 (22.1)	291 (17.6)	
	15–20	792 (45.6)	484 (29.2)	
	> 20	361 (20.8)	157 (9.5)	
Mesh position	Onlay	219 (12.6)	800 (48.3)	< 0.001
	Retromuscular	1,416 (81.6)	667 (40.3)	
	Intraperitoneal	88 (5.1)	162 (9.7)	
	Other	13 (0.7)	28 (1.7)	
Readmission (90 days)	Yes	342 (19.7)	285 (17.2)	0.067
Reoperation (90 days)	Yes	87 (5.0)	56 (3.4)	0.023
Recurrence	Yes	106 (6.1)	143 (8.6)	0.006

### Recurrence

In total, 249 (7.3%) patients underwent operation for hernia recurrence during follow-up. Patients who underwent operation for recurrence were significantly younger (58.5 years (13.4) vs. 60.7 years (12.9) *P* = 0.007). Significantly more of the patients who had recurrence had initial defect closure with non-absorbable suture compared to the group that did not have recurrence (57.4% vs. 48.2% *P* = 0.006). Mesh location also differed significantly: Patients with recurrence more frequently had an onlay or intraperitoneal mesh-repair compared to patients who did not have recurrence, where the retromuscular mesh position were most frequent (onlay: 36.1% vs. 29.5%, intraperitoneal: 10.8% vs. 7.1% and retromuscular: 50.2% vs. 62.3% *P* < 0.001). Patients with recurrence more often were readmitted or underwent reoperation within the first 90 days after repair compared to those without recurrence (readmission: 30.1% vs. 17.6%, reoperation: 11.2% vs. 3.7%, *P* < 0.001 for both). Sex distribution, type of incision, comorbidity score, and hernia defect and mesh size did not differ between the groups.

**Fig. 2:**
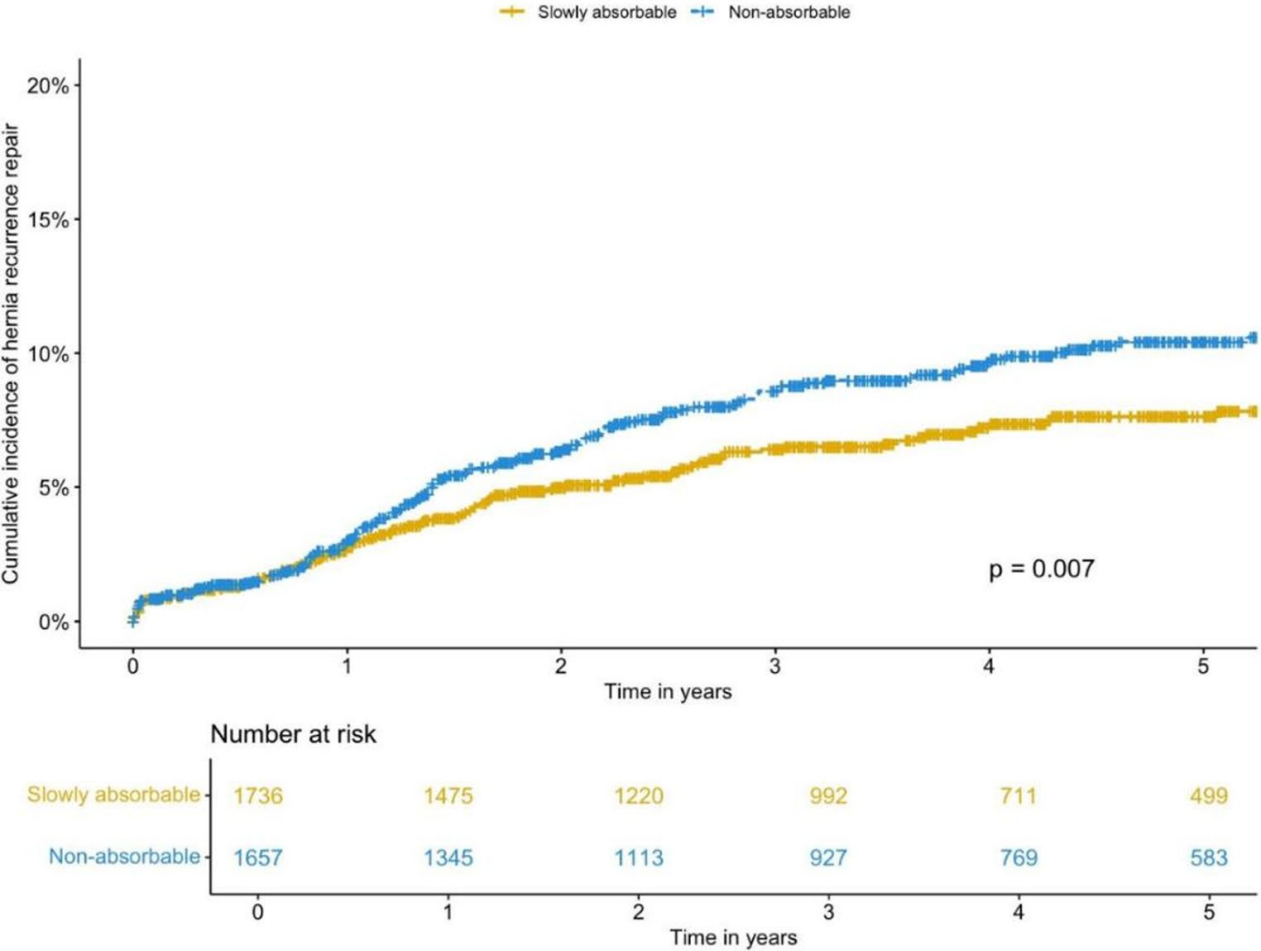
Cumulative incidence of reoperation for hernia recurrence according to type of suture used for defect closure

The overall 5-year cumulative incidence of operation for hernia recurrence was 9.0 (95% CI 7.86–10.13). According to type of suture, results showed that from one year after surgery there was a continuous temporally higher absolute risk of recurrence if non-absorbable suture had been used compared to slowly absorbable (5-year cumulative incidence: 10.4% vs. 7.6% *P* = 0.007, Fig. 2).

Multivariate cox regression analysis showed that non-absorbable suture was associated with a significantly increased risk of operation for recurrence compared to the slowly absorbable group (HR 1.33 95% CI 1.01–1.76 *P* = 0.043). Further, the risk of recurrence increased with increasing horizontal defect size (HR 1.04 (1.01–1.07) *P* = 0.006), recurrent compared to primary incisional hernia repair (HR 1.44 (1.07–1.93) *P* = 0.015), onlay mesh compared to retromuscular (HR 1.92 (1.40–2.65) *P* < 0.001), and vertical compared to horizontal incision (HR for the latter: 0.69 (0.51–0.93) *P* = 0.014). Sex, age, and comorbidity were not associated with increased risk of operation for recurrence. See Table 2.

**Table 2:** Cox proportional hazards multivariable regression analysis of the risk for reoperation for hernia recurrence (CCI = Charlson comorbidity Index, HR = Hazard Ratio)

Variable		HR	95% CI	*P*
Sex	*Female*	Ref		
	*Male*	1.03	0.80–1.33	0.817
Age		0.99	0.98–1.00.98.00	0.055
CCI	*0*	Ref		
	*1*	1.00	0.69–1.45	0.986
	*2*	0.88	0.61–1.27	0.493
	*> 2*	1.27	0.91–1.79	0.165
Index surgery	*Primary incisional*	Ref		
	*Recurrent incisional*	1.44	1.07–1.93	0.015
Orientation of incision	*Vertical*	Ref		
	*Horizontal*	0.69	0.51–0.93	0.014
	*Other*	0.66	0.43–1.02	0.059
Horizontal defect size		1.04	1.01–1.07	0.006
Mesh position	*Retromuscular*	Ref		
	*Onlay*	1.92	1.40–2.65	< 0.001
	*Intraperitoneal*	1.69	1.09–2.63	0.018
	*Other*	3.45	1.58–7.57	0.002
Suture type for fascial closure	*Slowly absorbable*	Ref		
	*Non-absorbable*	1.33	1.01–1.76	0.043

### 90-day reoperation

Univariate analysis showed that a CCI above 2 was significantly associated with 90-day reoperation among the total of 143 patients in this group (CCI > 2 35.7% vs. 24.6%, *P* = 0.019). Same analysis showed that patients who were reoperated had both significantly wider hernia defects and wider meshes (defect size: 8 (5.4) cm vs. 6.3 (4.6) cm, *P* < 0.001) compared to the patients who were not. Slowly absorbable sutures were more frequently used for the patients who underwent 90-day reoperation (60.8% vs. 50.7% *P* = 0.023) with significant differences in mesh positions, with higher count of retromuscular meshes in the group who underwent reoperation (71.3% vs. 61.0% *P* = 0.013). Finally, significantly more of the patients who were reoperated within 90 days after index surgery, subsequently underwent operation for hernia recurrence during the long-term follow-up (19.6% vs. 6.8% *P* < 0.001). However, when subjected to multivariate analysis controlling for variables such as CCI, age, and defect size, retromuscular mesh position and use of slowly absorbable suture were not significant risk factors for 90-day reoperation, whereas higher comorbidity index and larger defect size sustained a higher risk association as found univariately (CCI > 2: HR 1.68 *P* = 0.026 and defect size: HR 1.06 *P* = 0.002). See Table 3.

**Table 3:** Logistic regression analysis of the risk for reoperation within 90 days after index incisional hernia repair (CCI = Charlson comorbidity Index, OR = Odds Ratio)

Variable		OR	95% CI	*P*
Sex	*Female*	Ref		
	*Male*	0.88	0.63–1.25	0.484
Age		1.00	0.98–1.01	0.870
CCI	*0*	Ref		
	*1*	1.30	0.77–2.19	0.329
	*2*	1.04	0.62–1.74	0.896
	*> 2*	1.68	1.06–2.66	0.026
Index surgery	*Primary incisional*	Ref		
	*Recurrent incisional*	1.24	0.80–1.92	0.332
Orientation of incision	*Vertical*	Ref		
	*Horizontal*	1.02	0.68–1.54	0.909
	*Other*	1.66	1.00–2.73.00.73	0.048
Horizontal defect size		1.06	1.02–1.10	0.002
Mesh position	*Retromuscular*	Ref		
	*Onlay*	0.81	0.50–1.33	0.407
	*Intraperitoneal*	0.63	0.28–1.39	0.252
	*Other*	2.15	0.72–6.37	0.168
Suture type for fascial closure	*Slowly absorbable*	Ref		
	*Non-absorbable*	0.85	0.58–1.25	0.414

## Discussion

### Key results

This nationwide database study of patients undergoing elective, open incisional hernia mesh repair suggests an association between the use of non-absorbable suture for fascial closure and increased risk of subsequent operation for hernia recurrence during a 5-year follow-up period. Specifically, we found an instantaneous 33% higher risk compared to patients who had had fascial closure with slowly absorbable sutures after adjusting for relevant confounders. To our knowledge, this study’s design and aim has not been precedented, which implies substantial impact and methodological strength enabled by the registry design. The study’s findings are a result of nationwide data collection from patients and surgeons, which strengthens the external generalizability compared to a single-center setting or a single surgeon’s experience.

### Interpretation

Studies examining suture material and incisional hernia development have focused on primary closure after laparotomy and not recurrence after incisional hernia mesh-repair. Therefore, the literature discussed in this paragraph is based on primary laparotomy closure. A comprehensive review studied patients who had undergone any type of laparotomy and found no significant difference in risk of hernia development after one year according to type of suture [13]. Notably, this was after only one year, which in fact resembles the tipping point of the results of our study where the risk difference between the two groups was not seen until exactly after one year, which coheres to the literature of increasing recurrence risk over time [20]. The same review found a significant risk reduction regarding sinus and fistula tract development when absorbable sutures were used over non-absorbable, which importantly are events related to the wound healing, however, our data did not include these outcomes. Another review concluded equal effectiveness for fascial closure comparing slowly-absorbable with non-absorbable suture regarding outcomes of incisional hernia, wound dehiscence, suture sinus formation, peri-operative complications, and surgical site infections [21]. However, the included studies had shorter follow-up and covered various types of non-hernia laparotomy procedures with mass closure. This is an important distinction in comparison to our study since (1) the gold standard today for abdominal wall closure is the small-bites technique, and (2) follow-up should be at least three years to sufficiently evaluate the rate [12, 22, 23]. In another study, seven factors associated with increased rate of incisional hernia after midline abdominal incisions were identified, including former surgery for abdominal aortic aneurism (AAA) and bariatric reasons as only surgical risk factors, with no proven effect of suture type. The present study did not include surgical history, which would have been of relevance [15]. Contrary—yet with only six months follow-up—a prospective comparative study on patients who had undergone any type of elective abdominal incision found a significantly higher rate of incisional hernia in the group with non-absorbable suture, as well as persistent pain, burst abdomen and discharging sinus [24].

### Wound healing and infection

From a biomedical glance, an experimental rat model study compared PDS, Prolene and Vicryl and analyzed tissue inflammatory cell response and collagen presence, and found a favorable macrophage response in the PDS group suggesting this as a better choice by benefitting regenerative capacity of the abdominal wall, which supports the findings of our study [25]. This notion is important in context of what is known about wound healing and fascial regeneration. Suturing technique with equal force distribution, e.g. small-bite technique, is paramount to achieve a favorable collagen I to III ratio, which inherently is altered in patients with hernias and is detectable not only locally, but also systemically. These conditions provide theoretical support for our findings given that slowly absorbable suture appropriately promotes collagen metabolism [26, 27]. Further, tensile and holding forces of the suture also depend on technique of suturing aponeurosis only and avoiding fat and muscle tissue, however, data on this was not retrievable from the database, and very difficult to objectively assess.

One study compared rapidly absorbable Triclosan-coated suture (Vicryl plus) to slowly-absorbable suture and found a lower 36-month incidence of wound infections in the former group, but no difference in incisional hernia incidence [28]. This is slightly unexpected, since wound infection is a well-described high-impact risk factor for hernia development [6]. In the current study, we saw that more of the patients who developed recurrence had had a reoperation within the first 90 days, which was expected – we did, however, not assess the direct relation between these two events including type of suture used for the 90-day reoperation, which was a conscious methodological choice.

### Recurrence, age and mesh position

For the entire cohort there was an overall 5-year recurrence incidence of 9.0%, which notably, does not account for patients who had clinical recurrences and did not undergo reoperation. Database studies have showed a 5-year clinical recurrence incidence of approximately 10–23% depending of number of previous incisional hernia repairs, a range likely to cover the total number of recurrences in this present study [29, 30]. Regarding age, univariate analysis showed that individuals who underwent operation for recurrence were significantly younger, however, in adjusted analysis this effect disappeared. This proposes other factors as heavier risk drivers, such as non-retromuscular mesh position, increasing hernia defect size, previous repairs, and midline incisions, all in coherence with what was earlier determined internationally in guidelines for non-emergency repairs [6]. Notably, the risk of recurrence almost doubled if onlay mesh was used compared to a mesh placed retromuscularly. This is a convincing result, and supports what is currently recommended, even though certainty of evidence has been stated as very low with serious imprecision and high risk of bias [6]. Finally, a very recent registry study found that slowly absorbable sutures did not increase the risk of reoperation compared with non-absorbable sutures, regardless of mesh use after primary ventral hernia ≤ 4 cm, suggesting slowly absorbable as a safe option [31].

### Limitations

The observational nature of this study carries the inherent limitation of selection bias risk opposed to an optimally randomized design. The data did not offer details about suture materials, however, traditionally in Denmark, Prolene sutures have been used for non-absorbable, whereas PDS/Maxon/Monomax have been used as the slowly absorbable sutures, which compromises generalizability. Although quality-of-life is a crucial patient-reported outcome measure within hernia research, unfortunately it is not yet an outcome available in the database, which otherwise would have been of high interest to assess [6]. BMI and smoking history were only recently included in the database, factors that also would be highly relevant for this type of study as reports have found them to pose a risk for both short-term complications and long-term recurrence [32–34].

### Slowly absorbable suture: enough and “minimally invasive”?

In resumé, choice of suture is not standardized for open incisional hernia mesh repair and will often rely on surgeon’s preference and/or institution inventory. Traditionally, non-absorbable sutures were used, possibly linked to the era of non-mesh repairs. The findings of this study challenge the hypothesis that suture type is not important for mesh repair: rather, these results suggest that the suture effect has a repairing function together with the mesh, which is theoretically supported by the fact that wound healing is significantly affected by a systemically conditioned collagen metabolism. This is not intuitive from the acknowledged evidence that mesh repair is superior to suture repair, which has established the mesh—often placed in the retrorectus or retromuscular position—as the main operator in re-establishing abdominal wall strength and function [35–38]. The findings of this study could be supported by reported complications associated with non-absorbable sutures—e.g. suture sinuses and fistula tracts—possibly impairing fascial healing by disturbing collagen synthesis and theoretically inducing subsequent disruption of one or more of the abdominal wall layers, potentially leading to an increased risk of chronic pain, chronic mesh infection, hernia recurrence, and reoperation [39]. The suture needs to be durable enough for the mesh to implant in the tissue and for the fascia to heal: to be “minimally invasive”. For this purpose, a slowly absorbable suture is suggested as better and adequate compared to non-absorbable.

## Conclusion

The findings of this nationwide database study suggest that for patients undergoing elective open incisional hernia mesh-repair, the choice of suture type for fascial closure may impact the risk of long-term operation for recurrence. While the nature of this study design does not offer strong evidence, these results indicate that using a slowly absorbable suture compared to a non-absorbable suture is associated with a lower risk of subsequent operation for recurrence. To confirm this association, more studies including randomized trials are needed, which should address biomechanical perspectives, technique of closure, specific suture types, and patient-reported outcome measures such as quality of life.

## Data Availability

Data will be available on request.
